# Supplementary material for: Development of Dopant-Free N,N′-Bicarbazole-Based Hole Transport Materials for Efficient Perovskite Solar Cells
Source: Int J Mol Sci. 2024 Dec 6;25(23):13117. doi: 10.3390/ijms252313117 (PMC11642623; doi:10.3390/ijms252313117)
Supplement: Supplementary file 1 [file ijms-25-13117-s001.zip › ijms-3317023-supplementary.pdf]

Development of Dopant-Free N, N′-bicarbazole-based Hole Transport Material for Efficient Perovskite Solar Cells

Muhammad Adnan [1*], Hira Naz [2], Muzammil Hussain [2], Zobia Irshad [1], Riaz Hussain [2] and Hany W. Darwish [3]

[1] Graduate School of Energy Science and Technology, Chungnam National University, Daejeon, 34134, Republic of Korea.
[2] Department of Chemistry, University of Okara, 56300, Pakistan
[3] Department of Pharmaceutical Chemistry, College of Pharmacy, King Saud University, P.O. Box 2457, Riyadh 11451, Saudi Arabia
* Correspondence: adnan5750@gmail.com; adnan@cnu.ac.kr;

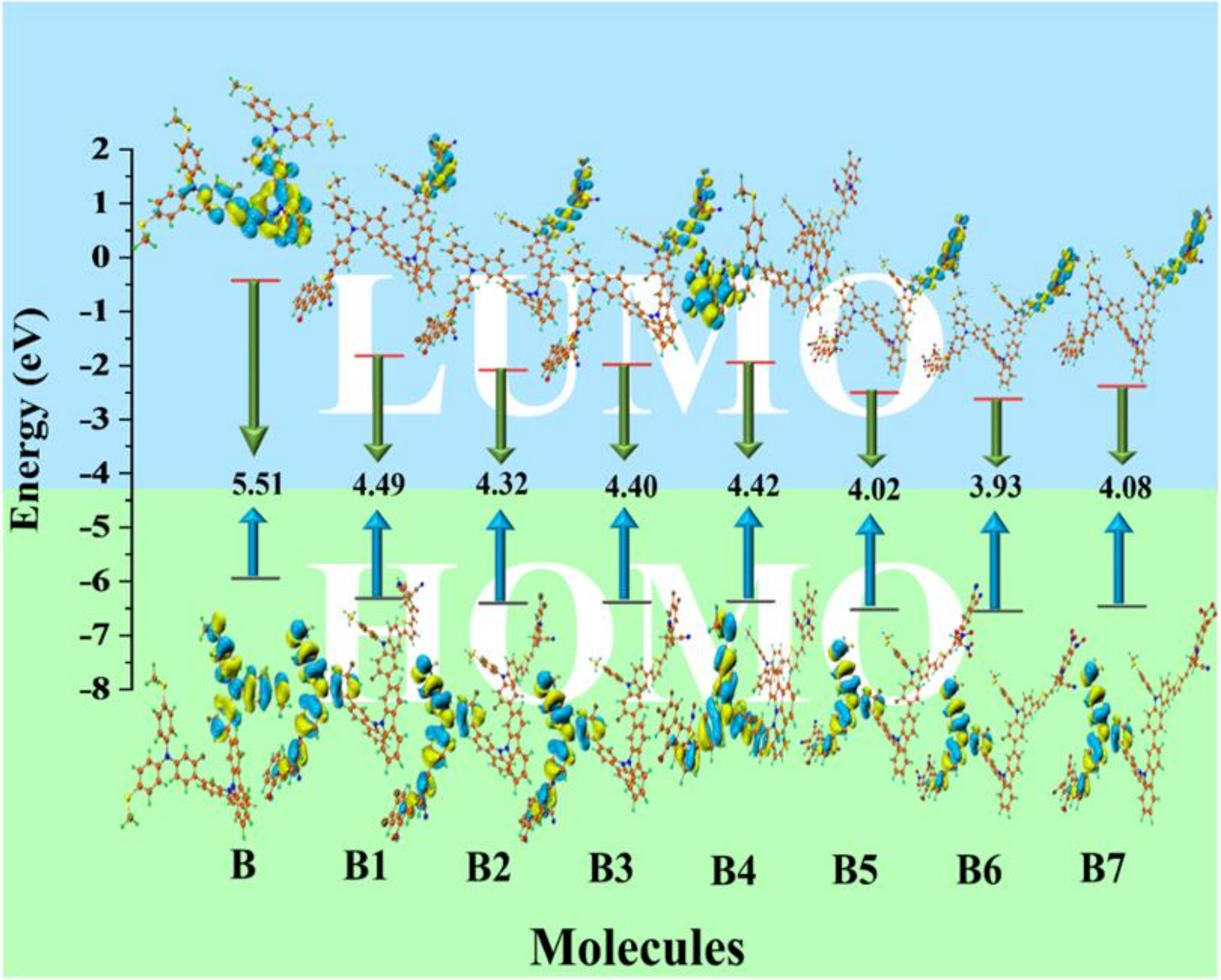

Figure S1: Dispersion of HOMO-LUMO charge density and computed energy gap of synthetic reference B and newly designed B1-B7 HTM series.

Table S1. Computed values of HOMO-LUMO and E_g of newly designed HTM series B1-B7 along with synthetic reference B.

**Molecules**	**(E_{HOMO}) (eV)**	**(E_{LUMO}) (eV)**	**$E_g = E_{LUMO} - E_{HOMO}$ (eV)**
**B**	-5.94	-0.43	5.51
**B1**	-6.31	-1.82	4.49
**B2**	-6.40	-2.08	4.32
**B3**	-6.38	-1.99	4.40
**B4**	-6.37	-1.95	4.42
**B5**	-6.53	-2.50	4.02
**B6**	-6.55	-2.62	3.93
**B7**	-6.46	-2.38	4.08
